# Novel extreme regression-voting classifier to predict death risk in vaccinated people using VAERS data

**DOI:** 10.1371/journal.pone.0270327

**Published:** 2022-06-29

**Authors:** Eysha Saad, Saima Sadiq, Ramish Jamil, Furqan Rustam, Arif Mehmood, Gyu Sang Choi, Imran Ashraf

**Affiliations:** 1 Department of Computer Science, Khawaja Fareed University of Engineering and Information Technology, Rahim Yar Khan, Pakistan; 2 Department of Software Engineering, School of Systems and Technology, University of Management and Technology, Lahore, Pakistan; 3 Department of Computer Science & Information Technology, The Islamia University of Bahawalpur, Bahawalpur, Pakistan; 4 Information and Communication Engineering, Yeungnam University, Gyeongsan, Korea; Hanyang University, KOREA, REPUBLIC OF

## Abstract

COVID-19 vaccination raised serious concerns among the public and people are mind stuck by various rumors regarding the resulting illness, adverse reactions, and death. Such rumors are dangerous to the campaign against the COVID-19 and should be dealt with accordingly and timely. One prospective solution is to use machine learning-based models to predict the death risk for vaccinated people and clarify people’s perceptions regarding death risk. This study focuses on the prediction of the death risks associated with vaccinated people followed by a second dose for two reasons; first to build consensus among people to get the vaccines; second, to reduce the fear regarding vaccines. Given that, this study utilizes the COVID-19 VAERS dataset that records adverse events after COVID-19 vaccination as ‘recovered’, ‘not recovered’, and ‘survived’. To obtain better prediction results, a novel voting classifier extreme regression-voting classifier (ER-VC) is introduced. ER-VC ensembles extra tree classifier and logistic regression using soft voting criterion. To avoid model overfitting and get better results, two data balancing techniques synthetic minority oversampling (SMOTE) and adaptive synthetic sampling (ADASYN) have been applied. Moreover, three feature extraction techniques term frequency-inverse document frequency (TF-IDF), bag of words (BoW), and global vectors (GloVe) have been used for comparison. Both machine learning and deep learning models are deployed for experiments. Results obtained from extensive experiments reveal that the proposed model in combination with TF-TDF has shown robust results with a 0.85 accuracy when trained on the SMOTE-balanced dataset. In line with this, validation of the proposed voting classifier on binary classification shows state-of-the-art results with a 0.98 accuracy. Results show that machine learning models can predict the death risk with high accuracy and can assist the authors in taking timely measures.

## Introduction

**Fig 1:**
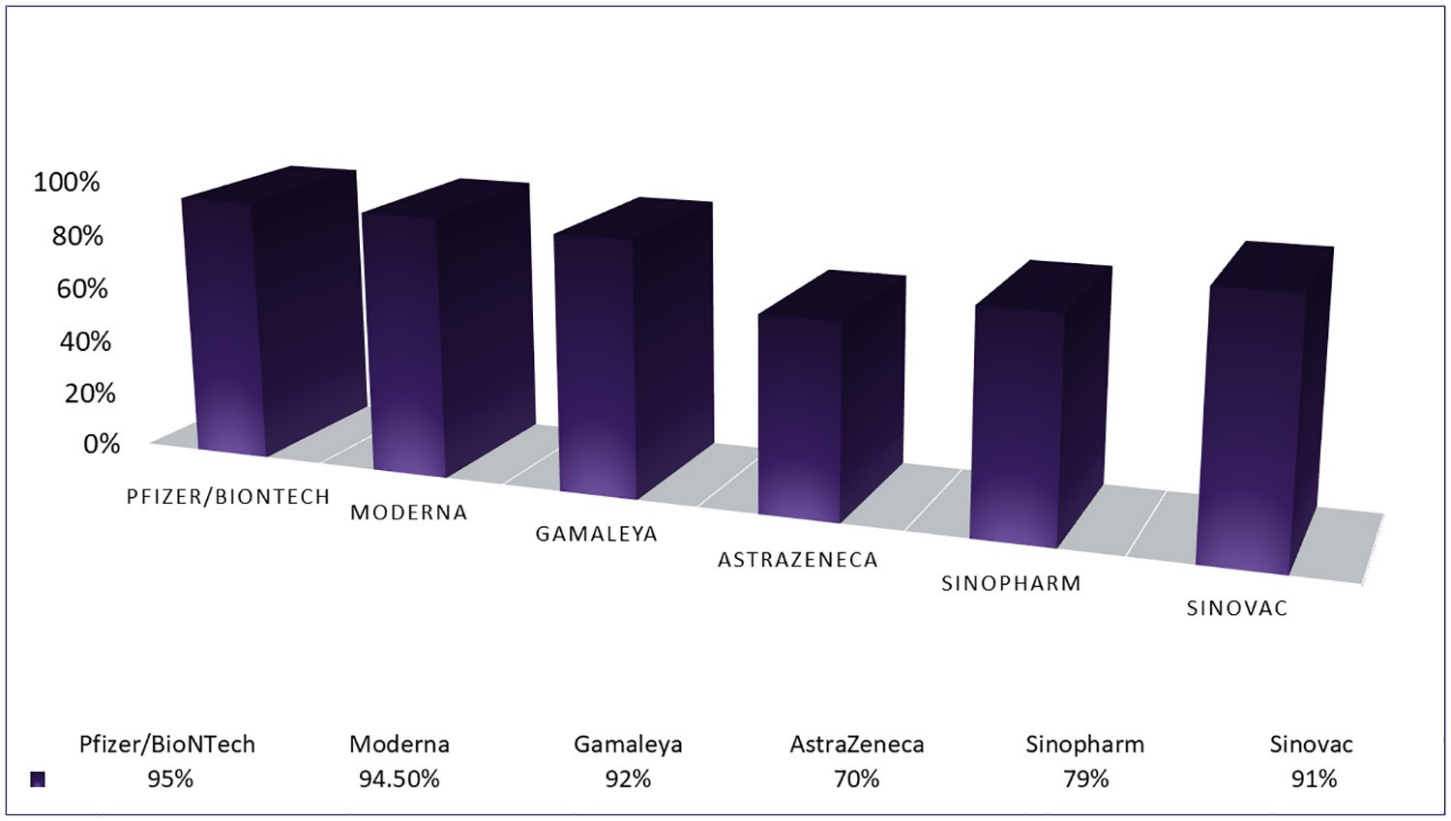
Vaccines and reported efficacy for COVID-19.

Several pandemics appeared during the last two decades like severe acute respiratory syndrome (SARS), Middle East respiratory syndrome (MERS), and corona virus disease 2019 (COVID-19), etc. COVID-19 led to infect approximately 308 million people in 223 countries leading and caused 5.492 million deaths as of 12 January 2020 [1]. The ongoing COVID-19 pandemic impacted the individual, as well as, public life of human beings on a global scale, and containing it seems to be very difficult. Although, it possibly can be confined like other viruses such as HKU1, NL63, 229E, and OC43, however, the substantial human and financial loss remains the main concern [2]. Precautionary measures against COVID-19 such as sanitation procedures, physical distancing, personal hygiene, mask usage, disinfection of the surfaces, and frequent hand washing are essential to reduce its spread. However, the case fatality ratio (CFR), a measure of mortality among infected cases, continues to increase [3]. Immunity against COVID-19 can facilitate a safe return to normal life [4] which is aimed by several developed vaccines like Moderna, Pfizer (BioNTech), Johnson & Johnson, etc. [5]. As of December 2020, several vaccines have been administered with different efficiency and immunity against COVID-19, as shown in Fig 1.

Vaccines do cause side effects, both minor and major, and COVID-19 vaccines have no exception. Reports of adverse side effects following the first and second doses of COVID-19 vaccination are submitted to the vaccine adverse event reporting system (VAERS). From January 1 2021 to March 19, 2021, a total of 5351 adverse events have been reported to VAERS. The adverse side effects range from mild to severe such as fever, pain, diarrhea, fatigue, blood pressure, chills, muscle pain, headache, and pain at the injection site. Similarly, several COVID-19 positive cases are reported in several countries even after full vaccination. Blood clotting, severe allergic reactions, cardiac problems, and resulting deaths are also reported following adverse events such as cardiac arrest, abdominal pain, etc. There is also a theoretical risk that vaccination could make infection severe by enhancing the respiratory disease [6]. Such adverse reactions and death reports make it significantly important to analyze the data regarding the adverse effects of COVID-19 vaccines and report reactions with a higher probability of fatality to assist healthcare professionals in prioritizing the cases with adverse effects and provide timely medical treatment.

Machine learning (ML) is the automated discovery of potentially valid or useful knowledge and novel hidden patterns from data [7]. ML models operate by identifying relationships and patterns among the data instances in single or multiple datasets. ML has been widely applied in healthcare sectors for its applications in simulating health outcomes, forecasting patient outcomes, and evaluating medicines [8]. Recently, ML has also been extensively used in the diagnosis and prognosis of many diseases like COVID-19, as immense data is being generated regarding COVID-19 [9]. Such data can be analyzed to predict the COVID-19 case and devise corresponding policies to contain the pandemic. Similarly, data associated with adverse events reports post-COVID-19, gathered by VAERS was made public on the 27th of January, 2021 which motivated current research.

This study demonstrates an enhanced ML-based prediction system to analyze the adverse events associated with the COVID-19 vaccine and predict individuals with symptoms that might cause fatality so that healthcare professionals can treat the individuals beforehand. It helps medical experts critically monitor vaccinated individuals with death risks. This study makes the following major contributions

- A systematic approach is presented for studying the adverse events reported after COVID-19 vaccination for possible death leading symptoms. Three significant events hold special importance in this regard including ‘not survived’, ‘recovered’, and ‘not recovered’.
- To obtain a higher prediction accuracy, a novel voting classifier ER-VC is devised that combines extra tree (ET) classifier and logistic regression (LR) under soft voting criterion. Multiple experiments are performed to investigate the problem using models like random forest (RF), LR, ET, multilayer perceptron (MLP), gradient boosting machine (GBM), AdaBoost (AB), k nearest neighbors (kNN), and stochastic gradient descent (SGDC). Moreover, long short term memory (LSTM), convolutional neural network (CNN), and bidirectional LSTM (BiLSTM) are also implemented for appraising the performance of the proposed approach.
- For reducing the effect of model overfitting and analyzing the influence of data balancing, synthetic minority oversampling technique (SMOTE) and adaptive synthetic (ADASYN) resampling approaches are also integrated.
- Performance of the proposed approach is compared in terms of accuracy, recall, etc., as well as, with the existing state-of-the-art studies.

The rest of this study is organized into five sections. Starting with the discussion of previous works related to this study, the study follows the proposed approach, ML models, and dataset description. After that, the analysis and discussion of the results are provided. In the end, the study is concluded.

## Related work

Substantial economic and human losses are inflicted by the COVID-19 pandemic around the globe. The disease is difficult to treat based on previous methods used for treatment. However, with the modern electronic health records and advanced technologies, conducting research has become fast and easy. Medical and government institutions maintain repositories of COVID-19 patients and the associated symptoms are used to explore health risks. Laboratory tests, radiological reports, and patients’ symptoms have been analyzed using ML models by many researchers. Early studies mostly focused on disease diagnoses and predicting the death rate of COVID-19 patients based on statistical models [10]. After some time, hospital records of patients are mostly used to identify potential risks [11].

The rapid outbreak and wide expansion of the COVID-19 pandemic and its potential risk to human lives compelled different medical research laboratories and pharma industries to start developing the COVID-19 vaccine at a fast pace. For providing herd immunity to people, there was a need for a safe and effective vaccine in a short time [12]. At the end of 2020, 48 vaccines were available at the clinical trial phase, and three vaccines including Pfizer, Moderna, and AstraZeneca completed this phase in the US [13]. During the first phase, millions of health professionals were vaccinated, then populations at higher risk such as people older than 65 years are covered [14].

Severe outcomes leading to the death risk of COVID-19 patients are associated with different pre-existing medical conditions and comorbidities [9, 15]. Approximately more than 40% of patients hospitalized with COVID-19 had at least one comorbidity [16]. In a similar study, authors analyzed comorbidities between survivor and non-survivor patients [17]. Common diseases included diabetes mellitus, cardiovascular disease, chronic obstructive pulmonary disease, hypertension, and kidney-related diseases. Various other biomarkers such as C-reactive protein, high level of ferritin, white lymphocyte count, blood cell count, procalcitonin, and d-dimer are related to health risks and are increasing the mortality rate of COVID-19 patients [18]. These biomarkers and other symptoms could offer advantages in predicting death risks.

Authors have explored many ML-based techniques using patients’ symptoms and laboratory reports during hospitalization [19]. Researchers are diligent in defeating COVID-19 by exploring ways of COVID-19 detection [20] and devising frameworks to control the spread of disease [21]. Researchers applied an ML model to electronic health records to predict the mortality rate of COVID-19 patients [22]. However, the non-infected population is getting benefits from vaccination. Because of heterogeneity among the population due to demographic categories, risk patterns regarding COVID-19 disease and vaccine are difficult to predict. Different factors are involved in predicting death risks such as unique health history, obesity, cancer history, hereditary diseases, and different immunity levels. Medical professionals are striving to allocate resources and provide help in maximizing the survival probability. This study makes a significant contribution toward maximizing the survival rate of vaccinated people by predicting the probability of fatal outcomes by analyzing the post-vaccination symptoms. We leveraged growing electronic records and advanced predictive analytical methods to predict the risk associated with the side effects of COVID-19 vaccines to assist healthcare professionals.

## Material and methods

The prime objective of this study is to provide a highly accurate prognosis for death-risk patients. In addition, recovered and not recovered cases are also considered concerning the adverse events reported after the second dose of the COVID-19 vaccine. This study considers a two-stage strategy where Stage I investigates the multiclass classification into ‘not survived’, ‘recovered’, and ‘not recovered’. Stage II, on the other hand, deals with the binary classification of the adverse reactions into ‘survived’ and ‘not survived’. A brief description of the dataset utilized in this study and the proposed methodology are discussed in this section.

### Dataset description

COVID-19 VAERS dataset acquired from Kaggle is used for experiments. VAERS is an open repository for benchmark datasets and has been used by several previous studies [23]. It contains reports of adverse reactions after the COVID-19 vaccine and records several other attributes related to the reporting individuals [24]. The dataset includes 5351 records and 35 variables; attributes and their description are provided in Table 1.

**Table 1:** Description of data attributes of COVID-19 world vaccine adverse reactions dataset.

Variable	Description
VAERS_ID	Identification number for each vaccinated case
RECVDATE	Receiving date of adverse reactions report
STATE	Region of the country from which report was received
AGE_YRS	Age of vaccinated individual
CAGE_YR	Age calculation of individual in years
CAGE_MO	Age calculation of vaccinated individual in months
SEX	Gender of vaccinated individual
RPT_DATE	Date on which report form was completed
SYMPTOM_TEXT	Reported symptoms
DIED	Survival status
DATEDIED	Date of death of vaccinated individual
L_THREAT	Severe illness
ER_VISIT	Visited doctor or emergency room
HOSPITAL	Is hospitalized or not
HOSPDAYS	Number of days individual was hospitalized
X_STAY	Elongation of hospitalized days
DISABLE	Disability status of vaccinated individual
RECOVD	Recovery status of vaccinated individual
VAX_DATE	Date on which individual was vaccinated
ONSET_DATE	Onset date of adverse event
NUMDAYS	ONSET_DATE-VAX_DATE
LAB_DATA	Laboratory reports
V_ADMINBY	Vaccine administration facility
V_FUNDBY	Funds used by administration to buy vaccine
OTHER_MEDS	Other medicines in use by vaccinated individual
CUR_ILL	Information regarding illness of individual at the time of getting vaccinated
HISTORY	Long-standing or chronic health-related conditions
PRIOR_VAX	Information regarding prior vaccination
SPLTTYPE	Manufacturer Report Number
FORM_VERS	Version 1 or 2 of VAERS form
TODAYS_DATE	Form completion date
BIRTH_DEFECT	Birth defect
OFC_VISIT	Clinic visit
ER_ED_VISIT	Emergency room visit
ALLERGIES	Allergies to any product

**Fig 2:**
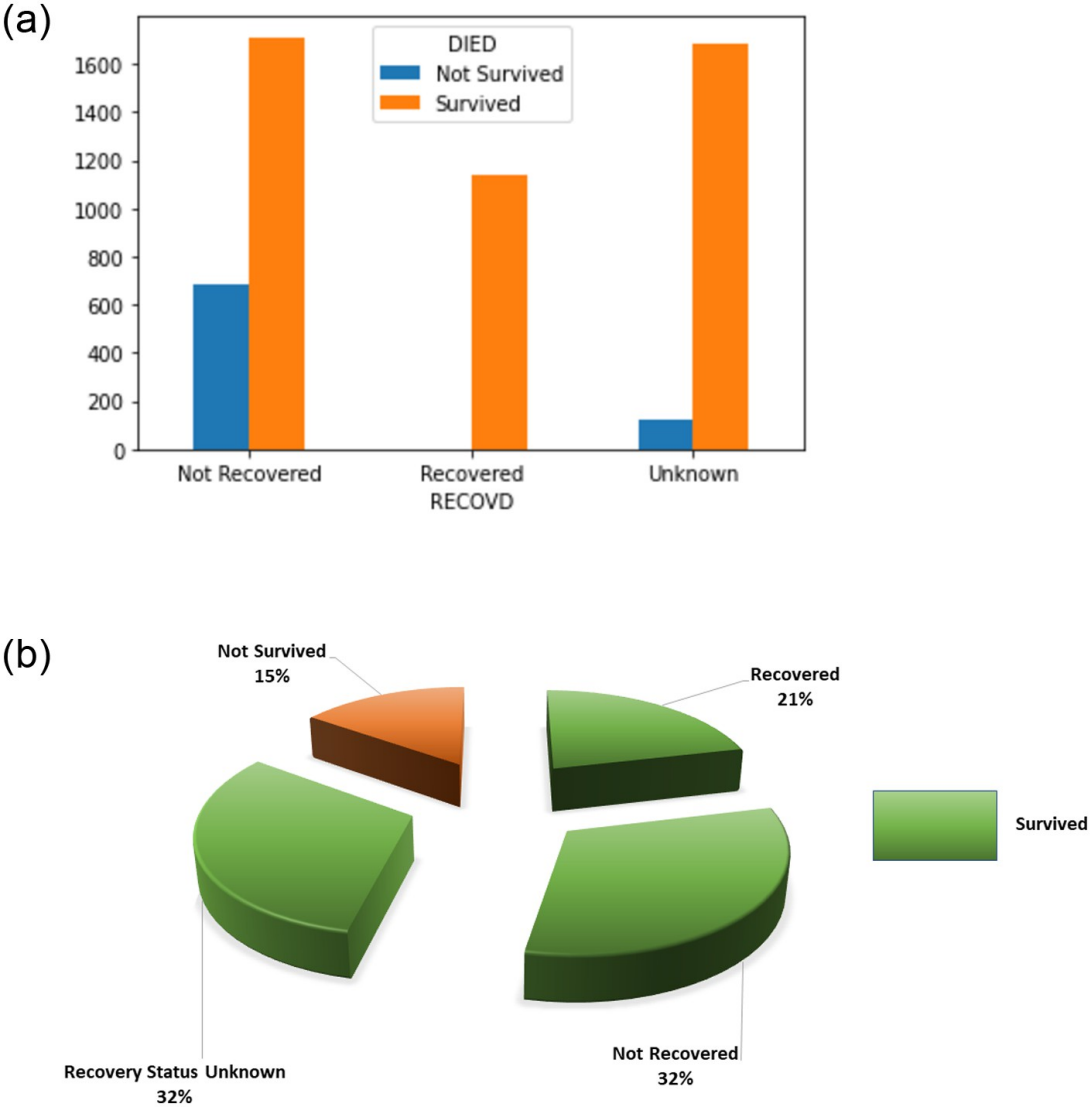
Dataset visualization, (a) correspondence between the categories related to ‘DIED’ and ‘RECOVD’ features, and (b) class distribution.

Since the goal is to investigate the death risk of vaccinated individuals, only three variables ‘RECOVD’, ‘DIED’, and ‘SYMPTOM_TEXT’ are utilized for multiclass classification and two variables ‘DIED’ and ‘SYMPTOM_TEXT’ for the binary classification problem. ‘DIED’ is further classified into ‘survived’ and ‘not survived’ which have 4541 and 810 records, respectively. ‘RECOVD’ has three target classes ‘recovered’, ‘not recovered’, and ‘recovery status unknown’ that have 1143, 2398, and 1810 records, respectively. A few of the ‘DIED’ cases are labeled as ‘not recovered’ while others as ‘recovery status unknown’ category, as shown in Fig 2a. Analysis of ‘DIED’ and ‘RECOVD’ features shows that a portion of the cases which did not recover from COVID-19 did not survive after the vaccination. Fig 2b shows that approximately 15% of the cases reporting adverse events died. The number of samples for different classes is not the same which makes the data imbalanced. For an unbiased and effective analysis, the records that correspond to ‘recovery status unknown’ are not considered except for the ones which belong to the ‘not survived’ category.

### Problem statement

The study considers the problem of individuals that receive the second dose of COVID-19 vaccination which can be Pfizer, Johnson & Johnson, etc. Despite minor side effects of the COVID-19, they are seldom reported for death. However, adverse reactions have a higher probability of serious illness leading to death. For notifying health care professionals beforehand and maximizing the survival rate of individuals facing adverse reactions, this study mines the adverse reactions of the COVID-19 vaccine reported to VAERS, for the prognosis of death risks.

### Proposed methodology

**Fig 3:**
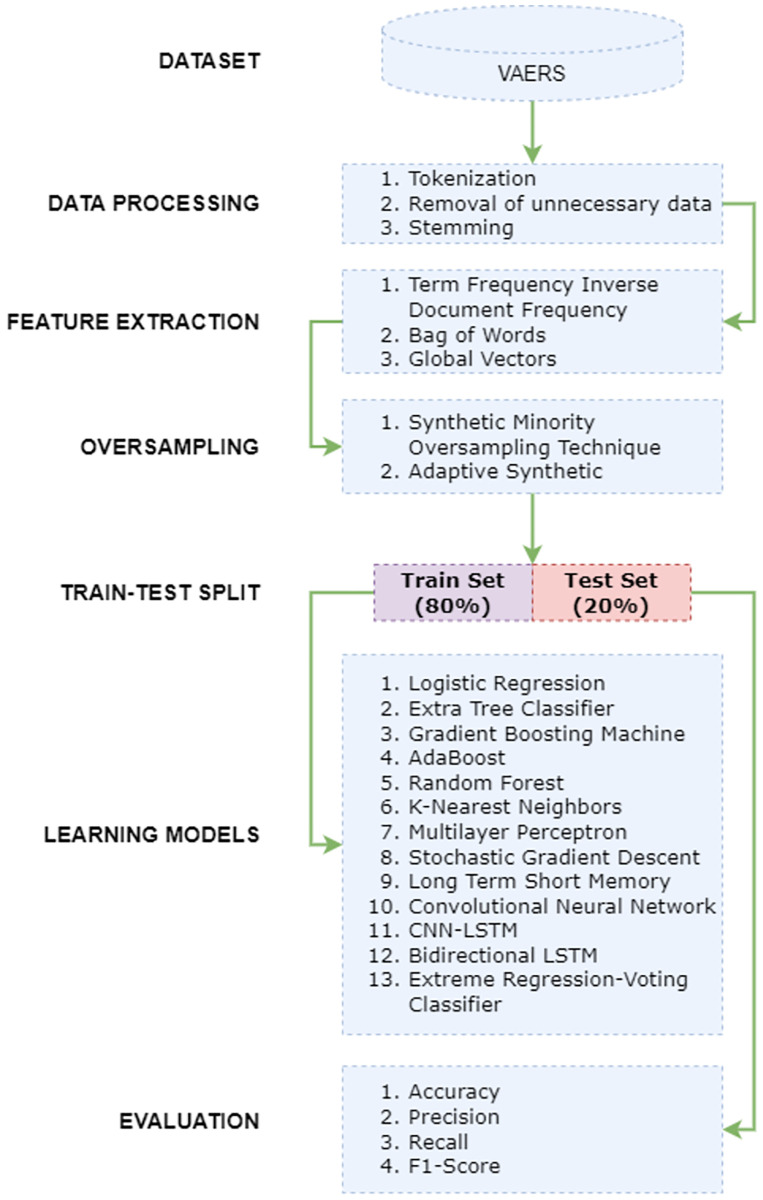
Architecture of the methodology devised for prognosis of death risks.

This study follows an ML-based approach for investigating the adverse events after the second dose of the COVID-19 vaccine. The architecture of the methodology used in this regard is given in Fig 3.

This study mainly follows multiclass classification which involves classifying adverse reactions as ‘not-survived’, ‘recovered’, and ‘not recovered’. In line with this, we integrated two data attributes including ‘RECOVD’, and ‘DIED’ as the target class, and one attribute ‘SYMPTOM_TEXT’ as a feature set in our experiments. The ‘RECOVD’ data attribute has three values including *Y* (recovered), *N* (not recovered), and *U* (recovery status unknown). We only utilized *Y* and *N* values from ‘RECOVD’ and *Y* values from ‘DIED’ for stage I experiments. This resulted in a total of 4351 instances out of which 810 instances correspond to the ‘not survived’ target variable, 2398 as ‘not recovered’, and 1143 instances are labeled as ‘recovered’. This shows the uneven distribution of target variables that can substantially dissipate the performance of classifiers. To overcome this problem, we oversampled the minority target variable using SMOTE and ADASYN.

To reduce the training and generalize the learning patterns for the classifiers, we integrated two feature extraction techniques including BoW (Bag of Words), TF-IDF (Term Frequency-Inverse Document Frequency), and GloVe (Global Vectors). Afterward, data is split into train and test sets with a ratio of 0.8 to 0.2. Furthermore, ML classifiers such as LR, ET, RF, GBM, AB, KNN, MLP, SGDC, and proposed voting classifiers learn the patterns regarding the target variable from the train set. Trained models are then tested on the unseen test data and evaluated under the criteria of accuracy, precision, recall, and F1 score.

### Data preprocessing

Data preprocessing aims at enhancing the quality of the raw input data to extract meaningful information from the input data. It is followed by the preparation of input data which includes cleaning and organization of the raw data to effectively build and train the ML-based classifiers. In the current study, various steps are taken to clean, normalize and transform the ‘SYMPTOM_TEXT’. We removed irrelevant data including punctuation, numeric, and null values from the input data. ML classifiers are prone to case sensitivity, for their efficient training we normalized the case of text by converting the text into lowercase. Afterward, we performed stemming using PorterStemmer(), and NLTK (Natural Language Tool Kit) function, for conversion of verbs into their root forms. As the last step of preprocessing, we removed stop words that are the most frequent in the text and are not significant for the classification.

### Feature extraction

Feature extraction is a technique that involves the extraction of significant and effective features from the preprocessed data for improved performance of predictive models on the unseen data. It follows the procedure of transformation of arbitrary data and finding features that are correlated with the target variable. ML classifiers guided by feature extraction technique tend to produce more accurate results [25]. Two feature extraction techniques including BoW, TF-IDF, and GloVe are utilized in this study.

BOW is the vectorization of text data into numeric features. It represents the word frequency within the text regardless of the information concerning its structure or position in the text. This technique considers each word as a feature [26]. It does not regard the number of times different terms appear in a document. A term’s presence in a corpus is the only factor that affects its weight.

TF-IDF quantifies a word in a document by computing the weight of each word which in turn shows the significance of a word in that text [27]. The weight is determined by combining two metrics, TF (Term Frequency) which is a measure of the frequency of a word in a document, and IDF (Inverse Document Frequency) which refers to the measure of the frequency of a word in the entire set of documents. Here document can be considered as ‘SYMPTOM_TEXT’ in the dataset. TF-IDF for frequency of a word *x* in document *y* can be computed as follows [28]:

$$\begin{array}{c} TF-IDF=\frac{f_{\left(x,y\right)}}{n_{y}}\times log\left(\frac{N}{df(x)+1}\right) \end{array}$$ (1)

where *f*_(*x*,*y*)_ is the frequency of word *x* in ‘SYMPTOM_TEXT’ records (*y*), *n*_*y*_ is the total number of words occurring in the *y*, *N* is the total number of ‘SYMPTOM_TEXT’ records, and *df*(*x*) is the number of ‘SYMPTOM_TEXT’ records in which word *x* is present.

GloVe generates word embeddings of the given ‘SYMPTOM_TEXT’ by mapping the relationship between the words. This is mainly done by aggregating the global co-occurrence matrices which provide information regarding the frequency of word pairs occurring together. Similar words are clustered together and different words are discarded based on the co-occurrence matrix of a corpus. Rather than training on the entire sparse matrix or individual context windows in a large corpus, the Glove model takes advantage of statistical information as exclusively nonzero elements in a word-word co-occurrence matrix [29].

### Data sampling

When a target variable is distributed unevenly in a dataset, it leads to a misleading performance by the ML models. The reason for this is that ML models learn the decision boundary for the majority class with more efficacy than the minority class. Therefore, showing poor performance in the prediction of minority class results in ambiguous and misleading results. Hence, changing the composition of an imbalanced dataset is one of the most well-known solutions to the problem of classifying an imbalanced dataset [30]. It can be done in two ways: undersampling or oversampling. Undersampling randomly reduces the majority class size and is mostly utilized when there is an ample amount of data instances whereas, oversampling arbitrarily duplicates the minority class and is effective when implemented on a small dataset. Since we have a limited number of records in our dataset, therefore, oversampling is the best fit for the proposed framework. One of the oversampling techniques is SMOTE [31] which is utilized in the current study.

SMOTE selects the data samples which are relatively close in the feature vector space and draws a line between those data samples [32]. It then generates synthetic data samples by finding *k* nearest neighbors for that particular data sample with *k* = 5. This results in simulated data samples that are comparatively at a close distance in the feature space from the data samples from the minority class.

ADASYN sampling is used to generate synthetic alternatives for each observation from the minority class. An observation from the minority class is ‘hard to learn’ if there are many instances from the majority class with features that are comparable to that observation. The concept of ‘appropriate number’ in this case is determined by how difficult it is to remember the initial observation. When drawn in the features space, a hard observation appears surrounded by features from the majority class. ADASYN is similar to SMOTE with one key difference, it biases the sample space towards locations that are not in homogeneous neighborhoods thus reducing the likelihood of any given point being chosen for duping.

### Machine learning classifiers

Supervised ML classifiers are utilized in this study for the prediction of target variables from the data. Implementation of ML classifiers is done in Python language using the ‘scikit learn’ module. ML classifiers are trained on data samples from the training set and tested using a test set that is unknown to the classifiers. ML classifiers integrated into this study are briefly discussed here and their corresponding hyperparameter settings are given in Table 2. GridSearchCV method is used to find the optimal parameters. For different parameters, the range has been determined using existing studies that report different parameters.

**Table 2:** Hyperparamter settings of supervised machine learning classifiers.

Model	Hyperparameter settings
RF	n_estimators = 100, random_state = 50, max_depth = 300
AB	n_estimators = 100, random_state = 50
LR	random_state = 50, solver = ‘saga’, multi_class = ‘ovr’, C = 3.0
MLP	random_state = 50, max_iter = 200
GBM	n_estimators = 100, learning_rate = 1, random_state = 50
ET	n_estimators = 100, random_state = 50, max_depth = 300
KNN	n_neighbors = 5
SGDC	max_iter = 2000, tol = 1e-3

#### Random forest

Random Forest is a tree-based ML classifier that integrates aggregated results obtained by fitting many decision trees on randomly selected training samples. The algorithm starts by using a split function to separate the initial set of training data into two disjoint sets; this process is recursive until a certain termination criterion is met, which leads to the generation of a leaf node. There is a probability distribution associated with each leaf node based on the number of voted labels as it reaches each leaf node. The random tree formation process generates a forest of random trees [33]. Each decision tree in RF is generated based on selection indicators such as Gini index, gain ratio, and information gain to select an attribute. It is a meta-estimator that can be used both for regression and classification tasks [34].

In random forests, when constructing an individual tree, not all features are taken into account, each tree is unique. Based on a different set of attributes and data, each tree is created independently. As a result, the CPU can be fully utilized to build random forests. The feature space is reduced since each tree does not take into account all the features. The feature space is significantly reduced since each tree does not take into account all the features. The voting/averaging method used by the algorithm results in stability [35].

In this study, different hyperparameters are used for the random forest algorithm to either make it faster or to boost the predictive power and its performance. Hyperparameter values for n_estimator, random_state, and max_depth are tuned according to the requirement. before averaging, the predictions parameter n_estimator states the number of trees the algorithm assembles. The value of the n_estimator parameter is set to 100 to get highly improved results, which are also said to be the number of weak learners implemented in the algorithm. Another parameter max_depth with the value of 300 is used to set the maximum depth level for each decision tree. These two parameters are used to enhance the prediction power of the algorithm by reducing the probability of overfitting and complexity in the decision tree. Lastly, the random_state parameter was used to control the randomness of the samples with the value of 50. If the model has a definite number of random_states it will produce the same outcomes and enhances the computational speed of the algorithm [36].

#### AdaBoost

AdaBoost also referred to as adaptive boosting is an iterative ensemble technique. Combining numerous weak learners into strong learners generates robust results. It is trained on weighted examples and provides optimized output by minimizing the error rate at each iteration [37]. An AdaBoost classifier starts by fitting a classifier on the original dataset as a meta-estimator and continues to fit additional copies of the classifier on the same dataset but adjusts the weights of poorly classified instances so that imminent classifiers focus more on complex scenarios [38].

Concerning the high accuracy rate, the AdaBoost algorithm is implemented with differently tuned hyperparameters including random_state, and n_estimator. In this study, the AdaBoost algorithm combines 100 weak learners to produce its final prediction so that the value of n_estimators is set to 100. The boosting process is stopped when it reaches the maximum number of estimators. The learning process is terminated early in the scenario of a perfect fit [39]. Regarding the random_state parameter, the value is set to 50. It states the randomness of samples during algorithm training which means that the random_state parameter limits the random selection of samples delivered at each boosting iteration of the model.

#### Extra Tree Classifier

Extra Tree Classifier is a collection of several de-correlated decision trees built from random sets of features extracted from training data. Each tree selects the best feature by computing its Gini Importance. ET incorporates averaging to control overfitting and enhance predictive accuracy [40].

The Extra Tree Classifier uses random subsets of features to build multiple trees and split nodes, but with two important differences: it does not bootstrap observations; implying that it samples without replacement, and nodes are split randomly rather than using the best split. Therefore, ET builds multiple trees by default with bootstrap = False, which means it samples without replacing. Nodes are split based on random partitions among a random subset of the features selected at each node. In Extra Trees, randomness arises from splits of each observation, rather than bootstrapping of the data.

This study used ET with different hyperparameter settings, where the n_estimator parameter with the value of 100 indicated the number of trees in the forest. The second parameter random_state used for the sampling of features to maintain or enhance the optimum split at each node with the value of 50. The splits for each of the max_features are drawn. Another parameter max_depth is implemented with the value of 300 which indicated the maximum depth of each tree in the forest [41].

#### Logistic regression

Logistic Regression is a statistical ML classifier that processes the mapping between a given set of input features and a discrete set of target variables by approximating the probability using a sigmoid function. The sigmoid function is an ‘S’-shaped curve that restricts the probabilistic value between the discrete target variables. It works efficiently for classification tasks [42]. The logistic regression technique is an advanced method of linear regression used for both classification and prediction in complex linear and non-linear datasets. A common application of this method is to model binary data. The logistic regression technique involves taking a given input value and multiplying it by a weight value [43]. Because of its defaulter detection ability, it has gained an incredible reputation in machine learning classifiers and is one of the simplest algorithms that can be applied to a wide range of classification problems. This is perhaps because it relies upon fewer assumptions [44].

This study used the LR algorithm with solver = ‘saga’ for computation as it works faster for large datasets and results were enhanced. The following parameter is multi_class which is used with the value ‘ovr’ because of its proficiency with binary classification. Then the inverse regularization parameter ‘C’ with the value of 3.0 is inversely positioned to the Lambda regulator and holds the strength of the regularization, reducing the chance of overfitting the model though smaller values reflect stronger regularization [45].

#### Multilayer perceptron

Multilayer Perceptron is an extensive feed-forward neural network that consists of three layers-input, output, and hidden layer. MLP works by receiving input signals which need to be processed at the input layer and performing predictions at the output layer. The hidden layer is the significant computational mechanism of MLP which is situated in the middle of the input layer and output layer. MLP is designed to map a nonlinear relationship between input and its corresponding output vector [46]. The back propagation learning algorithm is used to train the neurons in the MLP. MLPs can address problems that are not linearly separable and are designed to simulate any continuous function. Pattern classification, recognition, approximation, and prediction are some of MLPs most common applications [47].

In this study, MLP is implemented with different hyperparameters that include random_state and max_iter. The parameter random_state is used with its value set to 50 to determine a random number of generations for bias initialization and weights. The parameter max_iter is also implemented with the value of 200 to indicate the maximum number of iterations or to regulate the use of each data point [48].

#### Gradient Boosting Machine

Gradient Boosting Machine is a boosting classifier that builds an ensemble of weak learners in an additive manner which proves to be useful in enhancing the accuracy and efficiency of the learning model. Each weak learner in GBM attempts to minimize the error rate of the previous weak learner. It does so by integrating loss function with the gradients. It efficiently handles the missing values in the data [49]. GBM is a gradient-descent-based formulation of boosting approaches that are used to form a connection with the statistical framework. The learning mechanism in GBMs sequentially fits new models to offer a more precise estimation of the sample data. The basic idea behind this technique is to build new base learners that are maximally correlated with the loss function’s negative gradient, which is associated with the entire ensemble. Boosting algorithms, are reasonably easy to execute, allowing for experimentation with various model designs. GBM Computes the initial forecast by multiplying the new prediction by the learning rate [49].

In this study, the GBM is applied with hyperparameter elementary tuning to acquire high accuracy from the algorithm. The learning_rate is implemented with the value of 1.0 to reduce the contribution of each tree in the forest. The second parameter n_estimator is used to number the boosting stages to be completed that is set to the value of 100. Gradient boosting is relatively impervious to overfitting; therefore, a high number usually yields better results. Thirdly, at each boosting iteration, the random_state parameter is used to control the random seed assigned to each Tree estimator. It also regulates the random sequence of features at each split and manages the training data’s random splitting into a validation set [50].

#### K Nearest Neighbor

K-Nearest Neighbors is a straightforward ML classifier that maps the distance between a dependent variable and a target variable by adopting a particular number of k samples adjacent to the target variable. For classification, kNN predicts by considering the majority votes of the neighboring data points for the prevalent target variable [51]. The k-nearest neighbor algorithm is a non-parametric, supervised learning classifier that classifies or predicts the grouping of a single data point based on proximity. It can be used for both regression and classification tasks, however, it is most commonly employed as a classification technique, based on the idea that comparable points can be discovered nearby [52]. The kNN algorithm belongs to a group of “lazy-learning” classifiers, which means it just stores a training dataset rather than going through a training stage. This also implies that when a classification or prediction is being made, the entire computation takes place. It is also known as an instance-based or memory-based learning approach because it significantly relies on memory to retain all of its training data [53].

This study used the kNN algorithm with the ‘n_neighbors’ parameter that indicates the numbers of neighbors to use during the classification of each data point.

#### Stochastic Gradient Descent Classifier

The Stochastic Gradient Descent Classifier (SGDC) is an iterative method for locating the ideal smoothness qualities for an objective function e.g. differentiable or sub-differentiable. Because it replaces the actual gradient derived from the complete dataset with an estimate, it can be considered a stochastic approximation of gradient descent optimization that can be calculated from a randomly selected subset of the data [54]. SGDC is a modest and effective optimization approach for determining the values of parameters and function coefficients that maximize a cost function. In other terms, it is employed for discriminative learning of linear classifiers with convex loss functions. The update to the coefficients is conducted for each training instance rather than at the end of instances, which makes it effective for large-scale datasets. It minimizes the extremely high computational cost, especially in high-dimensional optimization problems, allowing for faster iterations in exchange for a reduced convergence rate [55].

SGD Classifier is employed with tuned hyperparameters including the ‘tol’ parameter with the value of 1e-3 it refers to be stopping criterion for the algorithm. Training will terminate after consecutive epochs if it is not None. Depending on the early stopping parameter, convergence is measured against the training or validation loss. Another parameter ‘max_iter’ is used to define the number of epochs that is 2000, the number of times the training data can be sent through.

### Proposed extreme regression-voting classifier

**Fig 4:**
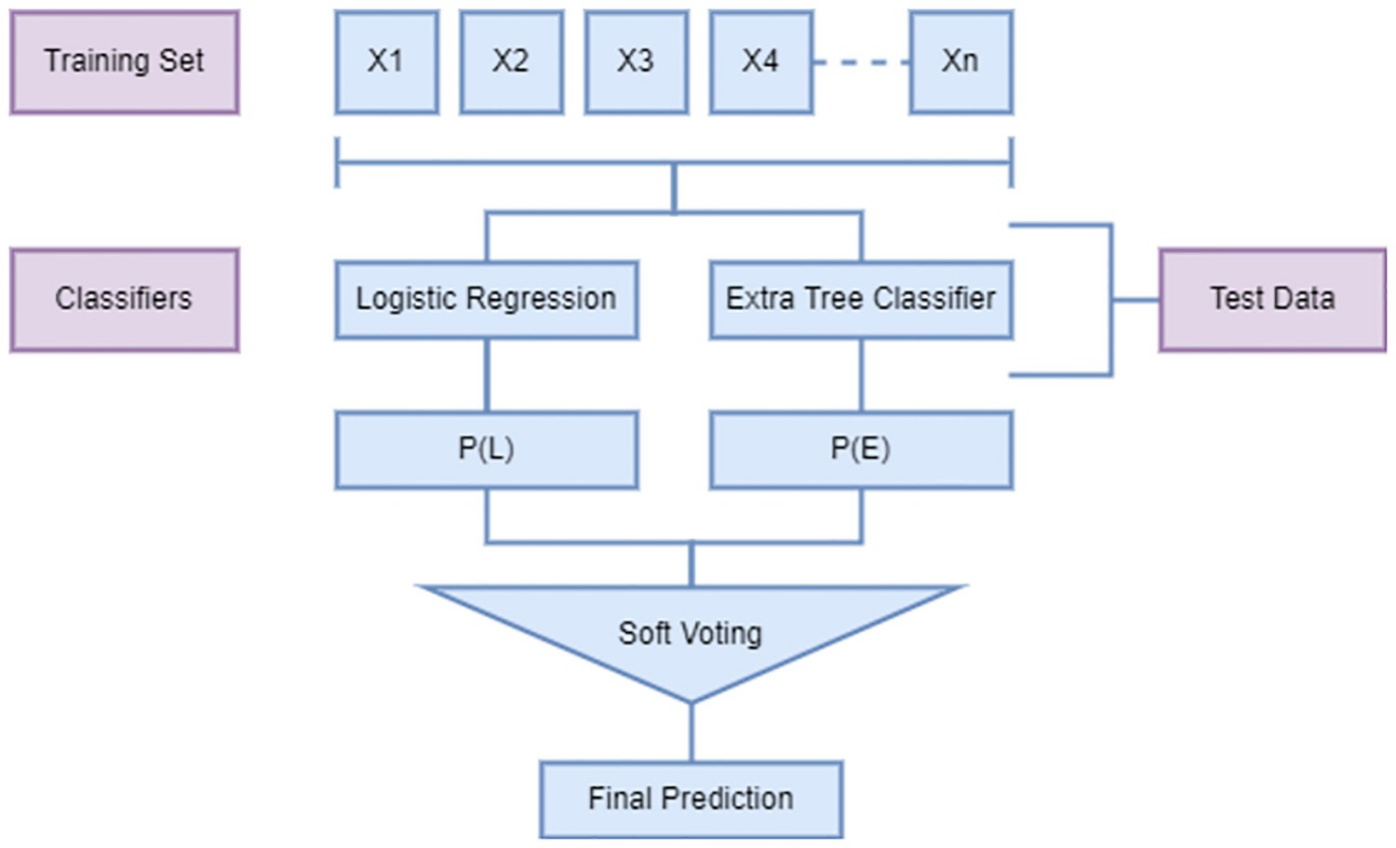
Framework of extreme regression-voting classifier.

ER-VC is a voting classifier that aggregates the output predictions of ET and LR to generate a final output. LR determines the significance of each feature of trained samples along with providing the direction of its association with less time consumption. This makes LR a good fit for our proposed voting classifier. Consequently, ET has been selected due to its randomizing property which restrains the model from overfitting. The foundation of the proposed classifier is building an individual strong model instead of discrete models with low accuracy results. It incorporates similar hyperparameter tuning of respected classifiers as described in Table 2. ER-VC is supported with soft voting criteria such that, it generates the final prediction by averaging the probability *p* given to the target class. The framework of the proposed ER-VC model is illustrated in Fig 4.

The working of the proposed ER-VC classifier is illustrated in Algorithm 1 [56]. We can compute the target class for the weights assigned to predictions *l*_1_, *l*_2_, *l*_3_, …, *l*_*n*_ made by classifier LR and *e*_1_, *e*_2_, *e*_3_, …, *e*_*n*_ by classifier ET respectively as

$$P\left(L\right)=l_{1},l_{2},l_{3},\ldots ,l_{n}$$ (2)

$$P\left(E\right)=e_{1},e_{2},e_{3},\ldots ,e_{n}$$ (3)

$$P_{f}=argmax\sum_{i=0}^{n}\left(P\left(L\right)+P\left(E\right)\right)$$ (4)

where *P*(*L*) and *P*(*E*) are the predictions made by *LR* and *ET*, respectively.

**Algorithm 1** Algorithm for proposed Extreme Regression-Voting Classifier (ER-VC)

**Input**: SYMPTOM_TEXT

**Output**: Vaccinated individual ← not survived or recovered or not recovered

 ***Procedure: Data Splitting***

  *Trn*_*s*_ = (*SYMPTOM*_*TEXT*, *Labels*)

  *Tst*_*s*_ = (*SYMPTOM*_*TEXT*)

  *Trn*_*s*_, *Tst*_*s*_

 ***Procedure: Voting Classifier***

  *V*_*c*_ = “*soft*”

  *L* = *LR*(*Trn*_*s*_)

  *E* = *ET*(*Trn*_*s*_)

 ***Procedure: Predictions made by L***

  *P*(*L*)←*Tst*_*s*_

  *P*(*L*) = *l*_1_, *l*_2_, *l*_3_, …, *l*_*n*_

 ***Procedure: Predictions made by E***

  *P*(*E*)←*Tst*_*s*_

  *P*(*E*) = *e*_1_, *e*_2_, *e*_3_, …, *e*_*n*_

 $P_{f}\leftarrow argmax\sum_{i=0}^{n}\left(P\left(L\right)+P\left(E\right)\right)$

### Performance evaluation metrics

For measuring the performance of a model, the selection of appropriate evaluation metrics is very important.

ML models are evaluated in terms of four basis outcomes true positive (TP), true negative (TN), false positive (FP), and false negative (FN). TP represents the correctly predicted positive instances, TN shows correctly predicted negative instances, FP is wrongly predicted positive instances, and FN is wrongly predicted negative instances. These metrics are further utilized to calculate accuracy, precision, recall, and F1 score. Following equations are used for this purpose

$$\begin{array}{c} Accuracy=\frac{TP+TN}{TP+TN+FP+FN} \end{array}$$ (5)

$$\begin{array}{c} Precision=\frac{TP}{TP+FP} \end{array}$$ (6)

$$\begin{array}{c} Recall=\frac{TP}{TP+FN} \end{array}$$ (7)

$$\begin{array}{c} F1-score=\frac{2\times recall\times precision}{recall+precision} \end{array}$$ (8)

## Results and discussion

For an in-depth evaluation of ML models and the proposed models, experiments should be exhaustive involving multiple scenarios and classes. This study follows two scenarios in this regard. First, ML models are trained to utilize three feature representation methods on an imbalanced, SMOTE-balanced, and ADASYN-balanced dataset. Accordingly, we selected the most relevant ML models to classify symptoms. Machine learning models include RF, LR, MLP, GBM, AB, kNN, ET, and, ER-VC. Experiments are performed to identify the most effective combination of feature extraction methods with ML models to classify symptoms into ‘recovered’, ‘not recovered’, or ‘not survived’.

### Experiments using imbalanced dataset

Initial experiments involve an imbalanced dataset using TF-IDF, BoW, and GloVe. Results of the proposed voting classifier are compared with the other baseline classifiers in terms of multiclass classification. Results presented in Table 3 show that LR achieves the highest results with a 0.73 accuracy score using TF-IDF on the imbalanced dataset. However, ER-VC and SGDC achieved a 0.72 accuracy score, which is the second-highest among all classifiers. It can be noticed that RF, ET, and MLP achieved a 0.71 accuracy value. Moreover, AB shows the worst result with a 0.64 accuracy value using TF-IDF on the imbalanced dataset. AB often cannot generalize well in the case of an imbalanced dataset.

**Table 3:** Classification results of machine learning models using TF-IDF on imbalanced dataset.

Models	Accuracy	Precision	Recall	F1 score
RF	0.71	0.70	0.71	0.70
AB	0.64	0.65	0.64	0.64
ET	0.71	0.70	0.71	0.70
LR	0.73	0.73	0.73	0.72
MLP	0.71	0.71	0.71	0.71
GBM	0.70	0.70	0.70	0.70
kNN	0.66	0.65	0.66	0.65
SGDC	0.72	0.72	0.72	0.72
ER-VC	0.72	0.72	0.72	0.71

Results presented in Table 4 indicate that using BoW as a feature representation method improves the results of most of the classifiers on the imbalanced dataset. From Table 4, it can be observed that BoW does not improve the performance of MLP and kNN. The proposed voting classifier, ER-VC achieves a 0.74 accuracy score using BoW which is 2% higher than what is achieved by TF-IDF using an imbalanced dataset.

**Table 4:** Classification results of machine learning models using BoW on imbalanced dataset.

Models	Accuracy	Precision	Recall	F1 score
RF	0.71	0.71	0.71	0.70
AB	0.68	0.69	0.68	0.68
ET	0.73	0.73	0.73	0.72
LR	0.72	0.72	0.72	0.71
MLP	0.71	0.71	0.71	0.71
GBM	0.73	0.73	0.73	0.72
kNN	0.52	0.55	0.52	0.50
SGDC	0.71	0.72	0.71	0.72
ER-VC	0.74	0.74	0.74	0.74

Table 5 shows the results of ML models when combined with GloVe features for the classification of an imbalanced dataset. A significant drop in the performance of ML classifiers can be observed. However, MLP yields the highest accuracy score of 0.65 whereas, the proposed ER-VC model does not perform well and acquired a 0.60 accuracy with GloVe features.

**Table 5:** Classification results of machine learning models using GloVe on imbalanced dataset.

Models	Accuracy	Precision	Recall	F1 score
RF	0.60	0.59	0.59	0.59
AB	0.57	0.55	0.55	0.54
LR	0.59	0.58	0.59	0.55
MLP	0.65	0.63	0.65	0.63
ET	0.61	0.59	0.59	0.58
GBM	0.57	0.57	0.57	0.57
kNN	0.55	0.54	0.55	0.54
SGDC	0.57	0.56	0.57	0.56
ER-VC	0.60	0.59	0.60	0.57

### Experiments using resampled dataset

The second scenario deals with the problem of imbalanced class distribution by the implementation of SMOTE and ADASYN. Data instances of the minority class are increased by oversampling to make a balanced dataset. Afterward, ML models have been trained using TF-IDF, BoW, and GloVe on SMOTE-balanced and ADASYN-balanced datasets.

#### Results of ML models on SMOTE-balanced dataset

The results of ML models using TF-IDF on the SMOTE-balanced dataset are presented in Table 6. It can be seen that SMOTE significantly improves the performance of ML models. As revealed by the results, SMOTE contributes to improving the models’ classification results, and 7 out of 9 models achieved higher than 80% results. SMOTE increases data instances of minority class by considering their distance to the *k* nearest neighbors of the minority class. In this way, the size of the minority class is increased by adding new data samples and making them appropriate for the training of the models. Hence the proposed voting classifier, ER-VC, which combines LR and ET outperforms other models and carries out prediction tasks with 0.85 accuracy, 0.85 precision, 0.85 recall, and 0.84 F1 score.

**Table 6:** Classification results of machine learning models using TF-IDF with SMOTE.

Models	Accuracy	Precision	Recall	F1 score
RF	0.81	0.82	0.81	0.81
AB	0.71	0.72	0.71	0.71
ET	0.82	0.83	0.82	0.82
LR	0.82	0.82	0.82	0.82
MLP	0.81	0.81	0.81	0.81
GBM	0.80	0.81	0.80	0.80
kNN	0.64	0.73	0.64	0.55
SGDC	0.82	0.82	0.82	0.81
ER-VC	0.85	0.85	0.85	0.84

Furthermore, the ML models are trained on the BoW feature representation technique. The performance of the models is compared in terms of classification results. Results shown in Table 7 prove that ML models using BoW do not achieve as robust results as achieved using TF-IDF on the SMOTE-balanced dataset.

**Table 7:** Classification results of machine learning models using BoW with SMOTE.

Models	Accuracy	Precision	Recall	F1 score
RF	0.78	0.79	0.78	0.78
AB	0.73	0.75	0.73	0.74
ET	0.78	0.78	0.78	0.78
LR	0.79	0.79	0.79	0.79
MLP	0.75	0.75	0.75	0.75
GBM	0.77	0.78	0.77	0.77
kNN	0.60	0.70	0.60	0.55
SGDC	0.76	0.76	0.76	0.76
ER-VC	0.81	0.81	0.81	0.81

Finally, ML models are combined with GloVe features for the classification of adverse reactions. The results reveal an overall decrease in the performance of ML models as shown in Table 8. However, a significant improvement in the results can be observed on the SMOTE-balanced dataset as compared to the performance of ML models when integrated with GloVe features on imbalanced data. Consequently, it proves that the BoW and GloVe feature representation techniques are not very effective in improving the performance of the models on the SMOTE-balanced dataset. However, SMOTE significantly improves the performance of ML models in classifying adverse events as ‘not-survived’, ‘recovered’, and ‘not recovered’.

**Table 8:** Classification results of machine learning models using GloVe with SMOTE.

Models	Accuracy	Precision	Recall	F1 score
RF	0.73	0.73	0.73	0.73
AB	0.58	0.58	0.58	0.58
ET	0.75	0.75	0.75	0.75
LR	0.60	0.59	0.60	0.59
MLP	0.65	0.67	0.65	0.69
GBM	0.63	0.63	0.63	0.63
kNN	0.64	0.64	0.64	0.63
SGDC	0.57	0.60	0.57	0.53
ER-VC	0.73	0.73	0.73	0.73

#### Results of ML models on ADASYN-balanced dataset

Rresults of ML models using TF-IDF with an ADASYN-balanced dataset are presented in Table 9. Results reveal that ADASYN also shows improvement in the results of ML models when compared with the results of an imbalanced dataset. It can be observed that RF, ET, MLP, and SGDC show even better results with ADASYN than the results obtained with SMOTE. ADASYN uses density distribution for generating synthetic data. It increases data samples by generating more data for minority classes and helps models in providing better training. For the proposed model ER-VC, ADASYN has shown slightly better results in terms of precision and F1-score than SMOTE. ER-VC achieves 0.85 accuracy, 0.86 precision, 0.85 recall and 0.85 F1 score.

**Table 9:** Classification results of machine learning models using TF-IDF with ADASYN.

Models	Accuracy	Precision	Recall	F1 score
RF	0.82	0.82	0.82	0.82
AB	0.71	0.72	0.71	0.71
ET	0.83	0.83	0.83	0.83
LR	0.82	0.83	0.82	0.82
MLP	0.83	0.83	0.83	0.83
GBM	0.80	0.81	0.80	0.80
kNN	0.63	0.75	0.63	0.54
SGDC	0.83	0.84	0.83	0.83
ER-VC	0.85	0.86	0.85	0.85

ML models are also trained on BoW features with an ADASYN-balanced dataset and the results are presented in Table 10. Results indicate that ML models show comparatively poor results with ADASYN-balanced dataset than SMOTE-based dataset when trained on BoW features. The performance of the ML models including the proposed ER-VC is degraded with ADASYN on BoW features.

**Table 10:** Classification results of machine learning models using BoW with ADASYN.

Models	Accuracy	Precision	Recall	F1 score
RF	0.74	0.73	0.74	0.73
AB	0.71	0.70	0.71	0.70
ET	0.75	0.74	0.75	0.74
LR	0.73	0.73	0.73	0.73
MLP	0.73	0.72	0.73	0.73
GBM	0.74	0.74	0.74	0.74
kNN	0.53	0.59	0.53	0.47
SGDC	0.75	0.75	0.75	0.75
ER-VC	0.78	0.77	0.78	0.77

Lastly, ML models are trained on GloVe features using ADASYN to predict adverse reactions. Results in Table 11 present that ML models show lower performance with ADASYN than SMOTE. Results reveal that using BoW and GloVe with ADASYN does not provide better accuracy as compared to BoW and GLoVe with SMOTE. ADASYN shows the best results using the TF-IDF technique.

**Table 11:** Classification results of machine learning models using GloVe with ADASYN.

Models	Accuracy	Precision	Recall	F1 score
RF	0.70	0.69	0.70	0.69
AB	0.52	0.52	0.52	0.52
ET	0.73	0.73	0.73	0.72
LR	0.54	0.53	0.54	0.52
MLP	0.62	0.61	0.62	0.61
GBM	0.62	0.61	0.62	0.61
kNN	0.62	0.62	0.62	0.61
SGDC	0.54	0.55	0.54	0.54
ER-VC	0.69	0.69	0.69	0.69

### Impact of feature extraction approaches on performance ML models

**Fig 5:**
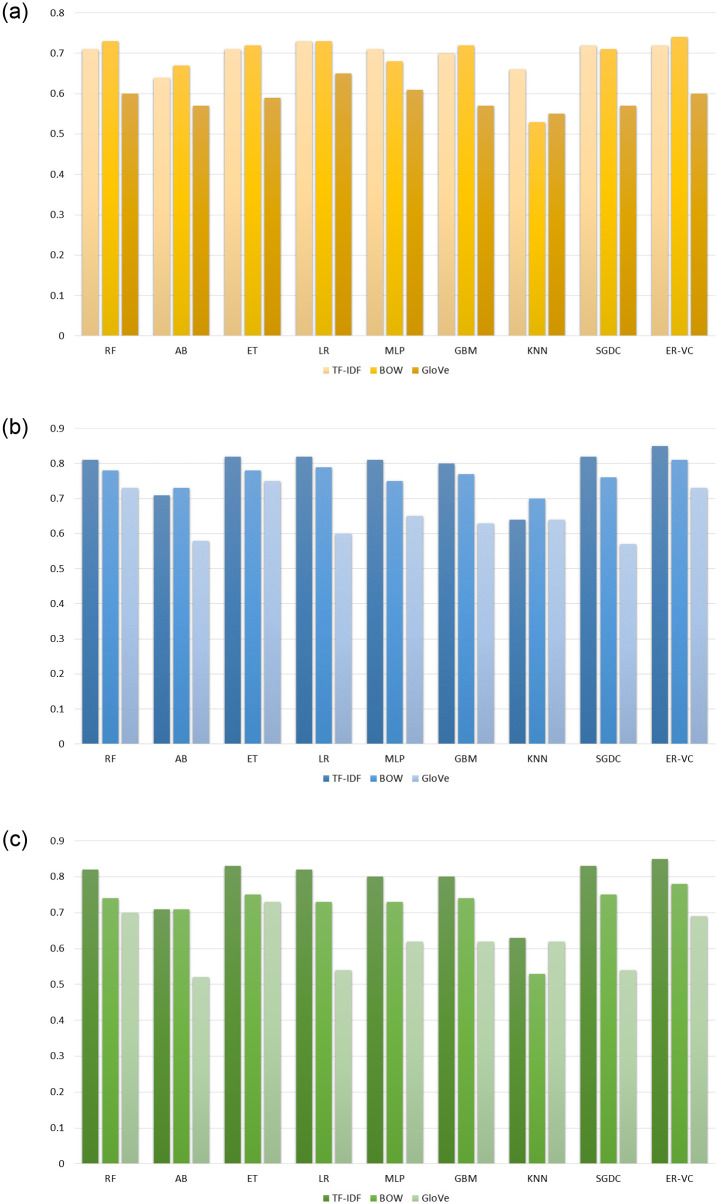
Performance analysis of ML models, (a) accuracy using TF-IDF, BoW and GloVe without SMOTE, (b) accuracy using TF-IDF, BoW and GloVe using SMOTE, and (c) accuracy using TF-IDF, BoW and GloVe using ADASYN.

Fig 5a presents the accuracy comparison of ML models using BoW, TF-IDF, and GloVe on imbalanced dataset while Fig 5b shows the performance comparison of ML models using BoW, TF-IDF, and GloVe using the SMOTE-balanced and ADASYN-balanced data. It can be observed that a substantial improvement in the accuracy of ML models occurred when they are trained using the over-sampled data with the TF-IDF feature set.

**Fig 6:**
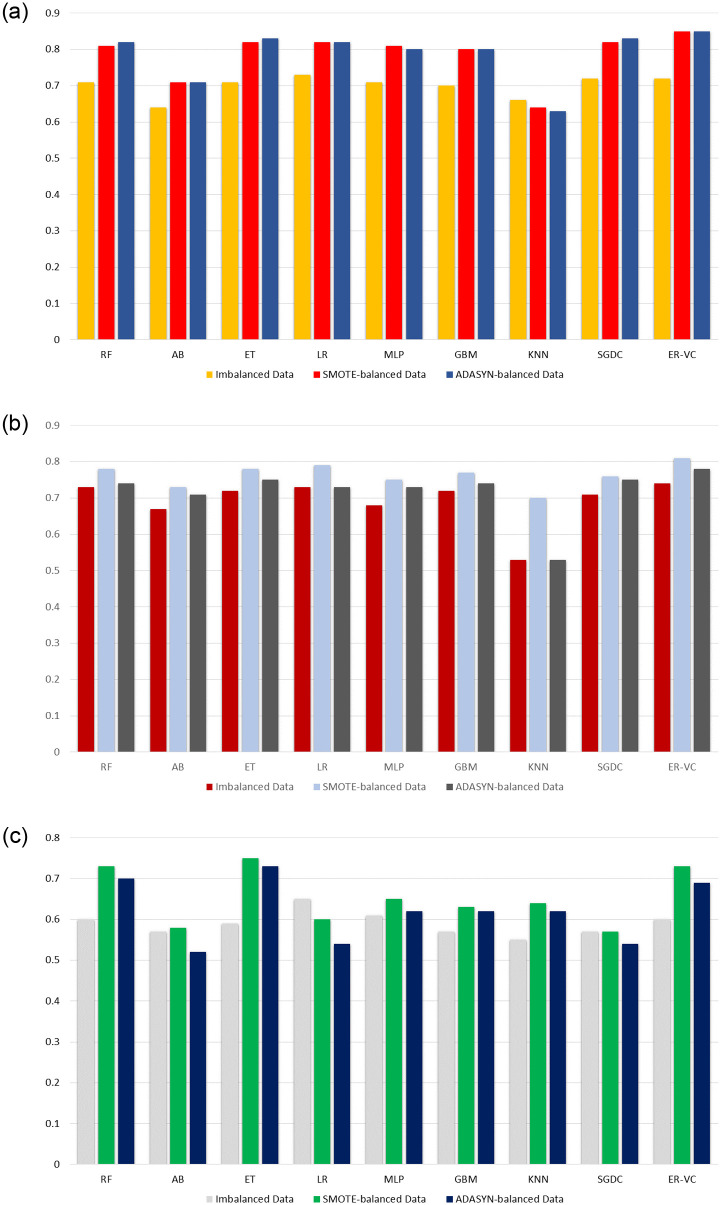
Performance analysis of ML models, (a) accuracy using TF-IDF with and without SMOTE, (b) accuracy using BoW with and without SMOTE, and (c) accuracy using GloVe with and without SMOTE.

Fig 6a presents the accuracy comparison of ML models using TF-IDF with and without oversampling techniques, Fig 6b presents the accuracy comparison of ML models using BoW with imbalanced and balanced data using SMOTE and ADASYN while Fig 6c shows the accuracy comparison of ML models using GloVe in the same scenario. It shows that the results obtained by using BoW on the balanced dataset are better than the results achieved by using BoW on the imbalanced dataset. On the other side, the results of the proposed model using BoW on the SMOTE-balanced dataset are 4% lower than the results obtained by using TF-IDF on the SMOTE-balanced dataset. While the accuracy result of the proposed model using BoW is 7% lower than the results achieved by using TF-IDF with the ADASYN-balanced dataset.

### Computational complexity of ML models

Table 12 shows the execution time taken by machine learning models for risk analysis. It indicates that the execution time varies with feature sets and learning models. All machine learning models take low execution time except for MLP and GBM. In terms of feature sets, machine learning models take less execution time than GloVe features whereas, TF-IDF features are second in line with BoW features taking the longest time. However, in terms of other evaluation parameters, TF-IDF features enable the models to make highly accurate predictions. The proposed ER-VC model performs slightly better with the ADASYN-balanced dataset however, the execution time is somewhat greater than the SMOTE-balanced dataset. This makes the proposed approach suitable to be used with SMOTE-balanced datasets with TF-IDF features.

**Table 12:** Execution time (seconds) of machine learning models.

Models	Imbalanced Data			SMOTE-balanced Data			ADASYN-balanced Data		
	TF-IDF	BoW	GloVe	TF-IDF	BoW	GloVe	TF-IDF	BoW	GloVe
RF	2.832	2.923	1.574	7.181	7.868	2.047	7.720	11.366	3.049
AB	3.135	2.635	1.805	5.935	3.809	2.174	14.161	6.512	3.693
ET	3.591	3.398	0.591	7.347	8.997	0.746	8.031	9.318	0.693
LR	0.329	2.001	0.353	0.482	1.297	0.398	0.705	2.507	0.619
MLP	50.243	76.554	9.629	63.849	144.615	5.300	97.751	150.671	6.951
GBM	15.773	8.580	7.658	27.473	19.028	15.259	40.384	14.226	11.475
kNN	0.180	0.212	0.148	0.345	0.307	0.192	0.485	0.301	0.338
SGDC	0.2997	0.132	0.292	0.387	0.297	0.115	0.147	0.151	0.129
ER-VC	8.145	4.308	0.642	7.712	10.223	1.863	9.447	10.624	1.023

### Performance comparison with deep neural networks

**Fig 7:**
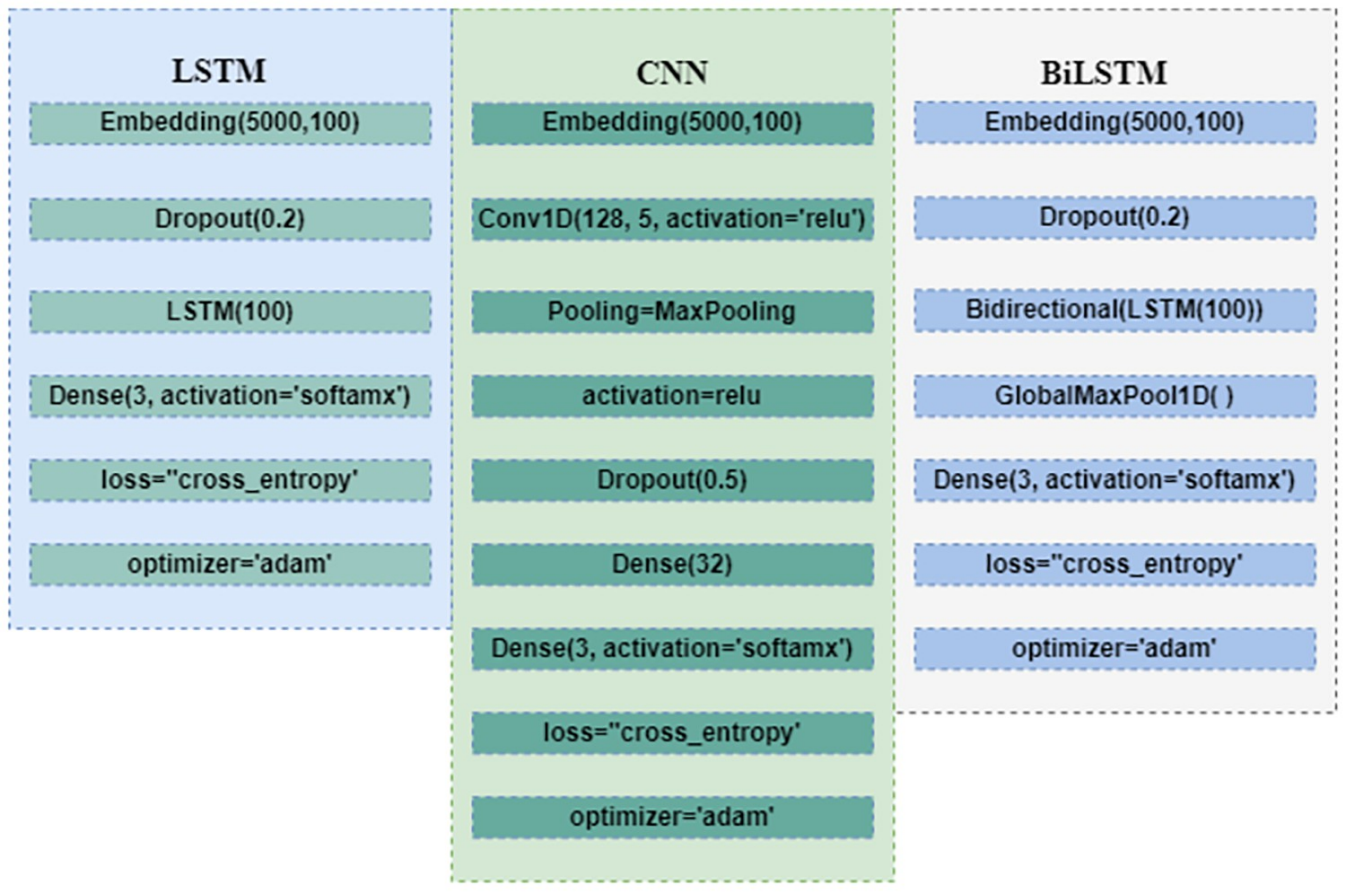
Layered architecture of the deep neural networks.

To substantiate the performance of the proposed voting classifier, it is also compared with deep learning models. We have used three deep learning models for experiments including LSTM [57], CNN [58] and BiLSTM [59] for comparison purposes. Layered architecture and hyperparameter values are presented in Fig 7. The architecture of these models is based on the best results and optimized hyperparameters.

**Fig 8:**
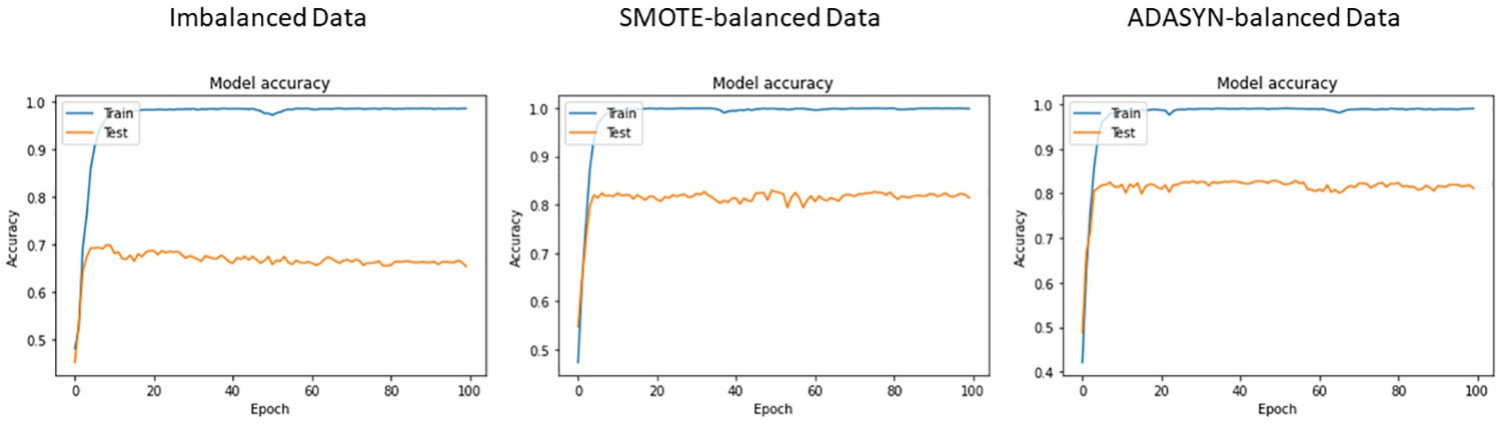
Accuracy measure of CNN with respect to each epoch.

**Fig 9:**
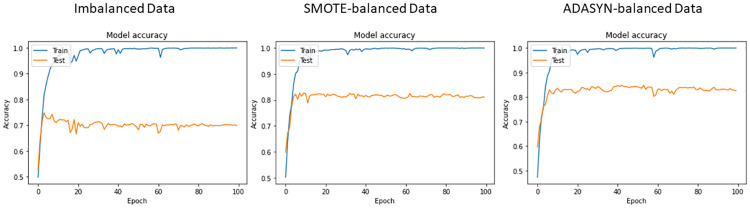
Accuracy measure of LSTM with respect to each epoch.

**Fig 10:**
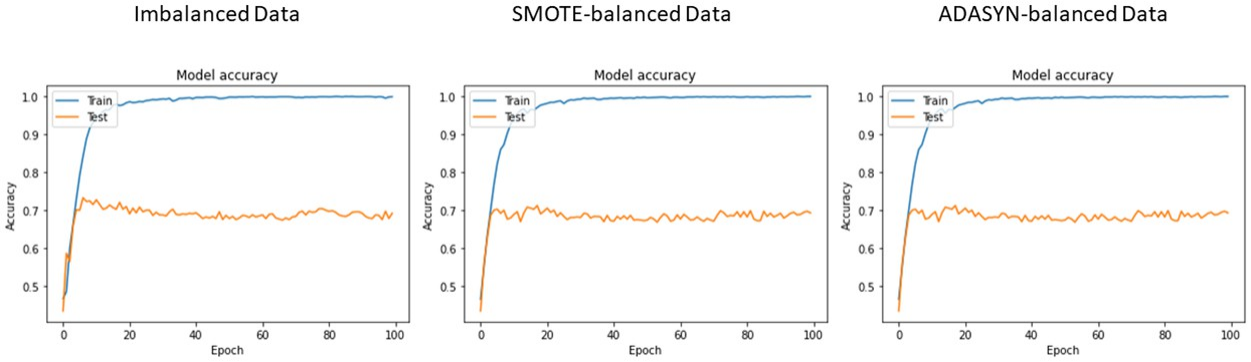
Accuracy measure of BiLSTM with respect to each epoch.

The same training and test split ratios are used for deep learning models. The deep learning models are used for experiments considering both the original, SMOTE-balanced, and ADASYN-balanced datasets. The training and testing accuracy curve of the used deep learning models including CNN, LSTM, and BiLSTM with balanced and imbalanced data are shown in Figs 8–10 respectively.

Classification results of deep learning models on balanced and imbalanced datasets are presented in Table 13. It can be observed that LSTM achieves the highest result with a 0.70 value of accuracy, precision, recall, and F1 score on imbalanced data while CNN has shown the lowest results. Given the small size of training data available for the deep neural networks, the performance is not good. However, using the ADASYN-balanced dataset, LSTM scores 0.83, and CNN yields 0.81 accuracy, precision, recall, and F1 scores. In terms of the SMOTE-balanced dataset, LSTM and CNN score 0.81 while BiLSTM scores the lowest with 0.69 accuracy. The performance of BiLSTM is not impacted by the oversampling techniques as the accuracy score remains the same in both cases. It can be observed that ADASYN balanced the dataset with highly correlated synthetic instances which makes the ADASYN-balanced data more effective for deep learning models. However, these values are lower than the proposed model ER-VC. Despite that, results for deep learning models confirm that oversampling has significantly improved the performance of LSTM and CNN models while BiLSTM has achieved similar results.

**Table 13:** Classification results of deep neural networks without SMOTE.

Dataset	Models	Acc.	Prec.	Rec.	F1
Original Data	LSTM	0.70	0.70	0.70	0.70
	CNN	0.64	0.65	0.65	0.64
	BiLSTM	0.69	0.69	0.69	0.69
SMOTE-balanced Data	LSTM	0.81	0.81	0.81	0.81
	CNN	0.81	0.81	0.81	0.81
	BiLSTM	0.69	0.69	0.69	0.69
ADASYN-balanced Data	LSTM	0.83	0.83	0.83	0.83
	CNN	0.81	0.81	0.81	0.81
	BiLSTM	0.69	0.69	0.69	0.69

### Validation of proposed approach

The current study validates the proposed ER-VC model by predicting the survival status of the vaccinated individuals. Following this, we integrated ‘SYMPTOM_TEXT’ as features and ‘DIED’ as the target class. It involves a total of 5351 data instances among which 810 are labeled as *Y* (not survived) and the remainder of the records are labeled as *N* (survived). The proposed ER-VC model is trained on 80% train data which is preprocessed and balanced using SMOTE. Experimental results after testing ER-VC on binary classification are shown in Table 14. Empirical results showed that the proposed ER-VC model manifested state-of-the-art performance in the prognosis of death risks by analyzing the adverse events reported to VAERS. Concerning the feature set, TF-IDF leads with a 0.98 accuracy score with its ability to extract features with more predictive information regarding target variables as compared to BoW which only provides a feature set of terms irrespective of their importance in the document, and GloVe which is inefficient when it comes to unknown words.

**Table 14:** Classification results of proposed ER-VC model for binary classification on SMOTE-balanced data.

Feature	Acc.	Class	Prec.	Rec.	F1
BoW	0.96	survived	0.97	0.96	0.97
		not-survived	0.96	0.97	0.96
		weighted avg	0.96	0.96	0.96
TF-IDF	0.98	survived	0.98	0.98	0.98
		not-survived	0.98	0.98	0.98
		weighted avg	0.98	0.98	0.98
GloVe	0.91	survived	0.93	0.89	0.91
		not-survived	0.89	0.93	0.91
		weighted avg	0.91	0.91	0.91

**Fig 11:**
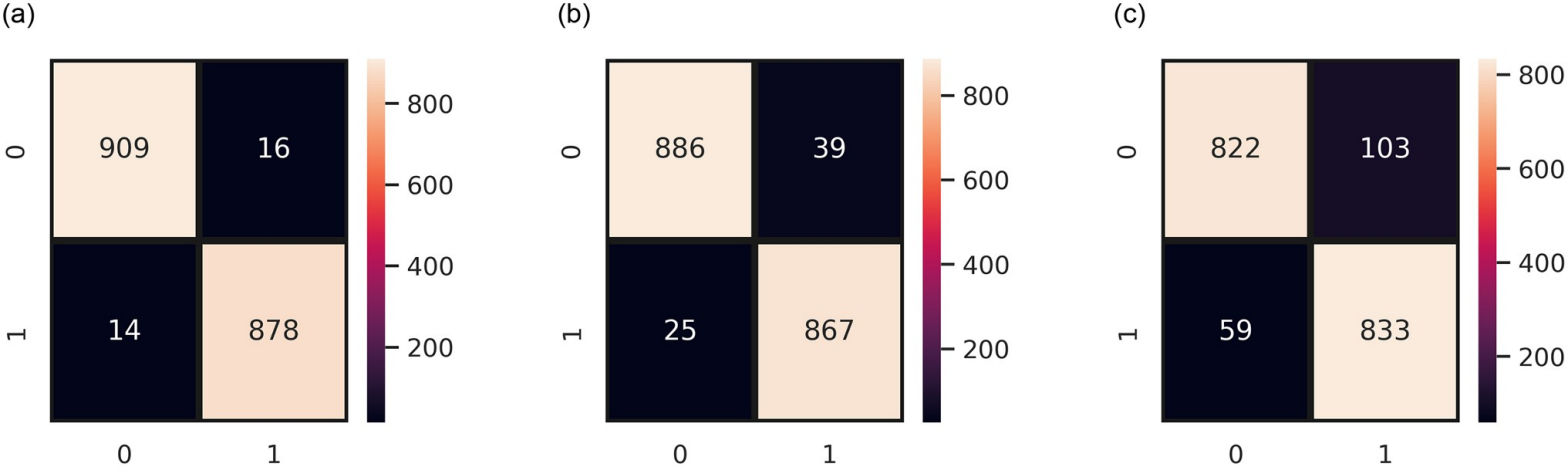
Confusion matrix of ER-VC concerning binary classification on SMOTE-balanced data, (a) ER-VC with TF-IDF, (b) ER-VC with BoW, and (c) RT-VC with GloVe.

Fig 11 demonstrates the number of instances predicted correctly following the given target variable. It can be observed that ER-VC wrongly predicted only 30 instances from a total of 1817 instances when integrated with TF-IDF features as shown in Fig 11a. Contrarily, Fig 11b shows that ER-VC in combination with BoW features made 64 wrong predictions out of 1817 instances. Whereas, in the case of GloVe features, the wrong predictions totals 162 which shows its poor performance in binary classification as presented in Fig 11c.

**Fig 12:**
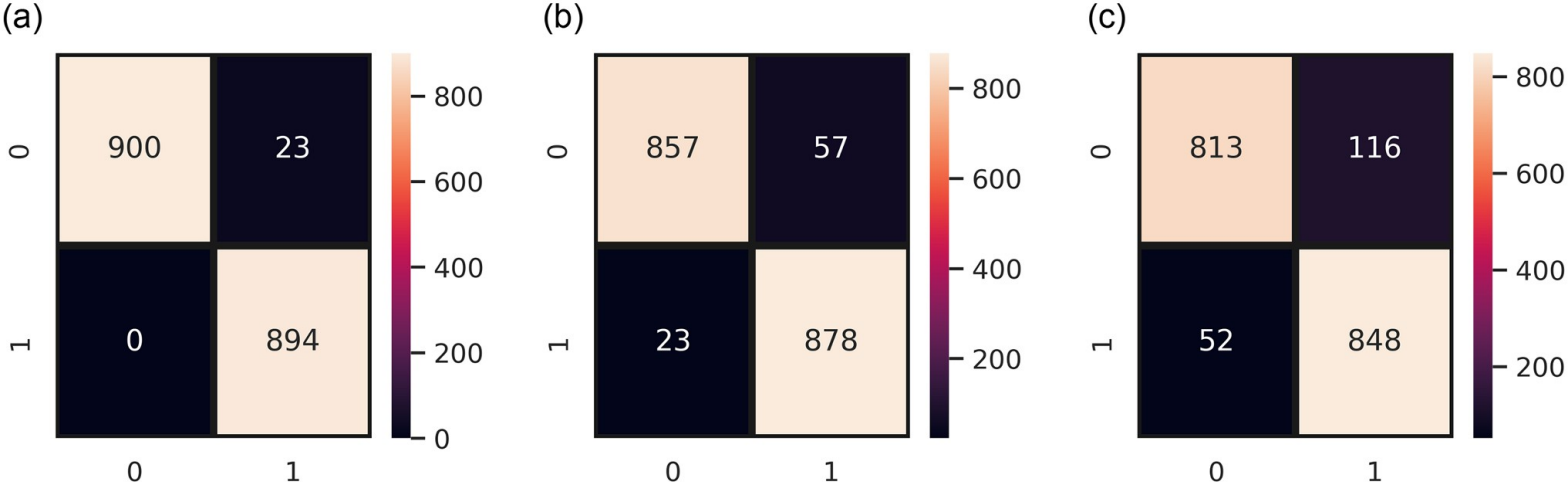
Confusion matrix of ER-VC concerning binary classification on ADASYN-balanced data, (a) ER-VC with TF-IDF, (b) ER-VC with BoW, and (c) RT-VC with GloVe.

Table 15 shows the results of proposed ER-VC model on ADASYN-balanced data. The results confirm the superior performance of ER-VC in combination with TF-IDF features. An accuracy score of 0.99 is obtained by the proposed model on ADASYN-balanced data that shows the effectiveness of the proposed model. It can be observed that TF-IDF features produced robust results in terms of SMOTE as well as ADASYN-balanced dataset. Fig 12 shows the number of correct and incorrect predicted instances by ER-VC on the ADASYN-balanced dataset. The efficacy of the proposed ER-VC in terms of TF-IDF features can be observed with only 23 wrongly predicted instances. This shows that in terms of binary classification in terms of TF-IDF features of the ADASYN-balanced dataset produced robust results. In a nutshell, BoW generates features irrespective of their importance concerning the target class whereas TF-IDF with its ability to extract features that are significant relative to the analysis excels in its performance. This resulted in an effective and robust prognosis of death risks following the COVID-19 vaccine using the proposed ER-VC model combined with TF-IDF features.

**Table 15:** Classification results of proposed ER-VC model for binary classification on ADASYN-balanced data.

Feature	Acc.	Class	Prec.	Rec.	F1
BoW	0.96	survived	0.97	0.94	0.96
		not-survived	0.94	0.97	0.96
		weighted avg	0.96	0.96	0.96
TF-IDF	0.99	survived	1.00	0.98	0.99
		not-survived	0.97	1.00	0.99
		weighted avg	0.99	0.99	0.99
GloVe	0.91	survived	0.94	0.88	0.91
		not-survived	0.88	0.94	0.91
		weighted avg	0.91	0.91	0.91

## Conclusion

The COVID-19 vaccines have been rolled out globally and allergic reactions after that are rare but post-vaccine side effects are being reported. Importantly, there is no doubt about the significance of vaccines in controlling the disease and preventing mortality during pandemics. This study promotes the vaccine and investigates the post-vaccine symptoms that lead to death and proposes an efficient soft voting classifier. This study will contribute in a way to assist health professionals in making effective strategies regarding COVID-19. The adverse events followed by the second dosage of the COVID-19 vaccine people are analyzed to predict three events that are ‘not survived’, ‘recovered’, and ‘not recovered’. Extensive experiments have been carried out using TF-IDF, BoW, and GLoVE with various machine learning models. In addition, the models are compared after balancing the dataset by applying SMOTE and ADASYN. The results of the models are compared with and without applying data balancing techniques. A significant improvement in the model’s performance can be observed with the balanced dataset. The proposed ER-VC model outperformed the remaining models with a 0.85 accuracy score when combined with TF-IDF feature set on the balanced dataset. An overall increase in performance of models is observed with SMOTE-balanced data as compared to the ADASYN-balanced dataset. Moreover, the comparison concerning the benchmark state-of-art deep neural networks confirms the performance of ER-VC is significantly better than deep learning models. We also noted that ADASYN performs better with TF-IDF features as compared to BoW and GloVe feature sets. Moreover, the effectiveness of the proposed model has been proved by experiments for binary classification where the model shows robust results with a 0.99 accuracy score for ADASYN-balanced data. In the future, we plan to direct our work to a union of feature extraction techniques to achieve improved results. Another possible future direction can be experimenting with a variety of vaccine adverse event datasets to broaden the scope of death risk analysis. We believe that the findingsare advocating the use of ML approaches for vaccine-related risks which can be used by health care professionals and health-providing services.
